# Comparative Analyses of the Conformational Dynamics Between the Soluble and Membrane-Bound Cytokine Receptors

**DOI:** 10.1038/s41598-020-64034-z

**Published:** 2020-05-04

**Authors:** Chao-Yie Yang

**Affiliations:** Department of Pharmaceutical Sciences, College of Pharmacy, University of Tennessee Health Science Center, Memphis, Tennessee United States of America

**Keywords:** Biochemistry, Biophysical chemistry, Cytokines, Lipids, Proteins, Structural biology, Biophysics, Computational biophysics, Drug development, Molecular dynamics, Computational chemistry

## Abstract

Cytokine receptors receive extracellular cues by binding with cytokines to transduce a signaling cascade leading to gene transcription in cells. Their soluble isoforms, functioning as decoy receptors, contain only the ectodomain. Whether the ectodomains of cytokine receptors at the membrane exhibit different conformational dynamics from their soluble forms is unknown. Using Stimulation-2 (ST2) as an example, we performed microsecond molecular dynamics (MD) simulations to study the conformational dynamics of the soluble and the membrane-bound ST2 (sST2 and ST2). Combined use of accelerated and conventional MD simulations enabled extensive sampling of the conformational space of sST2 for comparison with ST2. Using the interdomain loop conformation as the reaction coordinate, we built a Markov State Model to determine the slowest implied timescale of the conformational transition in sST2 and ST2. We found that the ectodomain of ST2 undergoes slower conformational relaxation but exhibits a faster rate of conformational transition in a more restricted conformational space than sST2. Analyses of the relaxed conformations of ST2 further suggest important contributions of interdomain salt-bridge interactions to the stabilization of different ST2 conformations. Our study elucidates differential conformational properties between sST2 and ST2 that may be exploited for devising strategies to selectively target each isoform.

## Introduction

The interleukin-1 (IL-1) family of cytokines and their receptors are key regulators of innate immunity that can initiate inflammatory response in hosts to fend off foreign antigens^1,2^. Ten IL-1 receptors (IL-1R) have been identified including the IL-1R1, IL-1R2, IL-1R accessory protein (IL-1RAcP or IL-1R3), IL-1R like 1 (IL-1RL1, ST2 or IL-1R4), IL-18Rα/β (or IL-1R4/7) and IL-1R accessory protein like 1 (IL-1RAPL or IL-1R9)^3,4^. IL-1R are single pass transmembrane proteins that contain an ectodomain (ECD) and a conserved cytoplasmic Toll-IL-1-Receptor (TIR) domain^2^. The ectodomain consists of three consecutive immunoglobulin-like C2 type-1,2,3 domains (denoted as D1-D3) connected by short linkers. The current model of the IL-1 pathway activation suggests that the IL-1 cytokine binds to its cognate IL-1R to recruit a second IL-1R member forming a hetero-trimeric protein complex and causing dimerization of TIR domains for downstream signaling^5^. Activation of the IL-1 pathway by extracellular cytokines can be regulated by negative or decoy receptors. The negative receptors, such as IL-1R2, lack the cytoplasmic domain to induce downstream signaling^6^. The decoy receptors include circulatory soluble receptors^7,8^ that sequester cytokines and limit the pool of free cytokines for binding to cytokine receptors on the cell membrane. The interplay of the binding between the cytokines and the membrane and soluble cytokine receptors allows to control the strength and duration of cytokine-mediated inflammatory response after cytokines are secreted to blood circulation.

Among the IL-1R members, ST2 is expressed on hematopoietic cells including T helper type 2 (Th2) cells, group 2 innate lymphoid cells (ILC2), regulatory T cells (Tregs) and mast cells^9,10^. Membrane-bound ST2 binds with the only known ligand IL-33 to recruits IL-1RAcP resulting in TIR domain dimerization between ST2 and IL-1RAcP^5,11^. Signal transduction via the ST2/IL-33 pathway leads to p38 MAP kinases phosphorylation and nuclear factor (NF)-κB activation^11^. Activation of the ST2/IL-33 axis in Th2 cells causes secretion of IL-4, IL-5, IL-13^12–14^ and IL-9^15^ that elicit type 2 immune response^16^. Dysregulation in the ST2/IL-33 signaling has been associated with several disease progression including excessive induction of ST2/IL-33 in Th2 cells^14^ found in asthma patients^17^. In patients developing graft versus host disease (GVHD) after hematopoietic cells transplantation (HCT), excessive increases of the sST2 level reduce the pool of IL-33^18^ for activation of the ST2/IL-33 axis in Th2, ILC2, and Tregs cells that leads to unrestrained inflammation in early GVHD progression^19–21^.

Antibodies^20,22^ and small-molecule inhibitors^23^ targeting membrane-bound and soluble ST2 have been reported. Both isoforms contain the same cytokine binding domains. This presents a challenge to develop specific inhibitors for use in different disease settings. Although antibodies therapeutics targeting the extracellular domains of cytokine receptors^22,24^ can recognize specific epitopes, no selectivity between the two forms has been reported. We^25^ and other groups^5,26^ have studied the conformations of the ectodomain of ST2 (ST2^ECD^) using Small Angle X-ray scattering (SAXS) and computational simulations. These data showed that ST2^ECD^ possess high conformational flexibility. A recent study indicated that ST2 undergoes a greater conformational motion than IL-1R1 before binding to the cognate cytokine on the cell membrane^5^. However, the extent of different conformational flexibilities between sST2 and ST2 remains unknown. Despite that sST2 and ST2 both contain the D1-3 domains, we hypothesized that ST2 may have limited conformational flexibility than sST2 because ST2 is fixated on the membrane via the transmembrane and the cytoplasmic domains. A better understanding of the differences between sST2 and ST2 conformations will provide insights to develop selective inhibitors.

**Figure 1:**
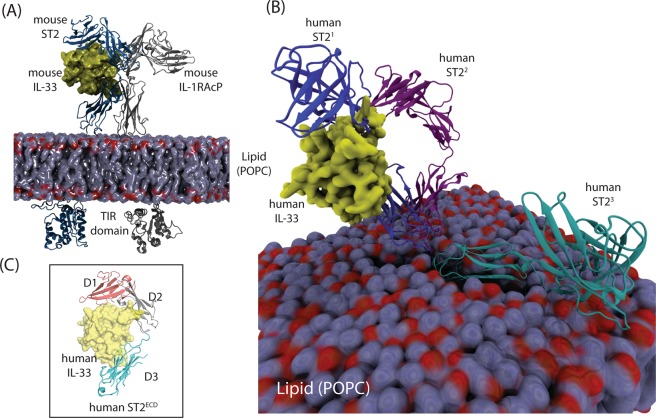
The crystal and the model structures of ST2 and sST2. (**A**) The model structure of mouse IL-1RAcP, mouse ST2, mouse IL-33 and the POPC membrane. (**B**) Models of human ST2^1^, ST2^2^ and ST2^3^. The lipid membrane built with ST2^3^ is shown and human IL-33 (yellow surface) bound to ST2^1^ is displayed as a reference. All ST2^1^, ST2^2^ and ST2^3^ are aligned to the transmembrane and the cytosolic domains in ST2^3^. (**C**) The crystal structure of human ST2^ECD^ and human IL-33 (PDBID: 4KC3). ST2^ECD^ is used to construct the sST2 model. The lipid membrane is shown as a quick surface model. Figures are prepared using PyMOL 2.3.0 (www.github.com/schrodinger/pymol-open-source) and VMD 1.9.4 (www.ks.uiuc.edu/Research/vmd)^27,28^.

In this work, we performed MD simulations of sST2 and ST2 in their glycosylated forms using the physiological salt concentration. A broad conformational space of sST2 was mapped using conformations obtained from conventional MD and accelerated MD (cMD and aMD) simulations. Because aMD was not implemented to sample ST2, we performed cMD simulations using three different orientations of ST2^ECD^ on the membrane (Fig. 1) to map the conformational space of ST2^ECD^ (or ST2 hereafter). We first employed the principal component analysis (PCA) to determine the conformational differences between sST2 and ST2. We then built the Markov State Model (MSM) to calculate the kinetics of conformational transition in sST2 and ST2. We showed ST2 exhibits less conformational mobility by visiting a more confined conformational space and at a slower timescale of transition. Pairs of salt-bridge interactions were found to contribute to the stabilization of the relaxed ST2 conformations.

## Methods

### Protein structures used in this study

Eleven crystal structures of protein complexes containing the ectodomain of the IL-1R members were used in this study (PDB entries: 4DEP^29^, 4GAF^30^, 3O4O^31^, 1G0Y^32^, 1IRA^33^, 1ITB^34^, 4KC3^26^, 5VI4^5^, 4R6U^35^, 3WO4^36^, 3WO3^36^). They included IL-1R1/IL-1β (PDBID:1ITB), IL-1R1/IL-1β/IL-1RAcP (PDBID:4DEP), IL-1R2/IL-1β/IL-1RAcP (PDBID:3O4O), IL-1R1/IL-1Ra (PDBID:1IRA), IL-1R1/EBI-005 (PDBID:4GAF), IL-1R1/AF10847 (PDBID:1G0Y), human ST2/IL-33 (PDBID:4KC3), mouse ST2/IL-33/IL-1RAcP (PDBID: 5VI4), IL-18R1/IL-18 (PDBID: 4R6U,3WO3) and IL-18R1/IL-18/IL-18RAPL (PDBID:3WO4). EBI-005 is an IL-1β chimera and binds more potently to IL-1R1 than IL-1β^30^. The crystal structure of ST2 extracted from ST2/IL-33 (PDBID: 4KC3) was used to construct sST2 (sequence:A18-P323) in which missing amino acids^25^ were rebuilt using the MOE program^37^. To construct membrane-bound ST2, a homology modeling of the transmembrane and cytoplasmic domains of ST2 (sequence: K321-A556) was performed using the iTasser server^38^. Five homology models were obtained and four models gave helical conformations in the transmembrane domain. We selected the first model because the N-terminal segment (K321-H326) of the transmembrane domain had the least clashes with the membrane. The segment (K321-P323) in ST2 from the crystal structure was then superimposed with the same segment in the homology model of the transmembrane and the cytoplasmic domains of ST2 to obtain the ST2 model (sequence: A18-Q556). The same procedures were used to build the transmembrane and the cytosolic domains of mouse IL-1RAcP and mouse ST2 complex using the crystal structure of the ectodomain of mouse IL-1RAcP, mouse ST2 and mouse IL-33 (PDBID: 5VI4)^5^.

### MD simulation setup

In preparation of the simulations, we have used the MOE program^37^ to determine the protonation state of ionizable groups on sST2 and ST2 under the standard physiological condition. The crystal structure of ST2 (only the ectodomain was resolved) showed three N-acetylglucosamine modified asparagines (N95, N140, N151) and five disulfide bonds. These post-translational modifications were retained in the MD simulations. The crystal structure of human ST2 was used as the initial conformation for sST2. For the initial conformation of membrane-bound ST2, we have set the backbone dihedral angle of L324 at 61°, 68° and 129° to generate three different orientations of ST2^ECD^ (denoted ST2^1–3^) relative to the lipid bilayer. The ectodomain of ST2^2^ deviated from that of ST2^1^ only by 7° whereas a significant backbone rotation of L324 in ST2^3^ from that of ST2^1^ was made. To prepare the sST2 and ST2 systems for simulations, we used the CHARMM-GUI server^39^. sST2 was solvated in a rectangular waterbox with the edge distance set to 10 Å and under 0.15 M of KCl salt concentration. To prepare the ST2 system, the transmembrane domain of ST2 was first oriented using the Orientations of Proteins in Membranes (OPM) server^40^. The transmembrane domain of ST2 was then inserted in a POPC lipid bilayer using the CHARMM-GUI server. The x- and y-dimension of the lipid bilayers were 140, 187 and 182 Å in length for ST2^1–3^ and the number of POPC molecules on the top and the bottom lipid layer were ST2^1^ (228,236), ST2^2^ (275,283), ST2^3^ (275,267) respectively. The thickness of the water layer was set at 85 Å on both the top and bottom layers. The KCl salt concentration was set at 0.15 M. After the system preparation, we found that the ectodomain of ST2^3^ was close to the lipid membrane surface. Based on the settings, ST2^3^ conformation will allow us to study how the close contact between the ectodomain of ST2 and lipid affects the motion of the ST2 ectodomain.

The CHARM36 additive force field parameters^41^ were used for both sST2 and ST2. The force field parameters for glycan and POPC were obtained from Glycan Reader^42^ and Membrane builder^43^ in CHARMM-GUI server. The TIP3P model^44^ was used for the water molecule. The force field parameters of the systems were converted into the AMBER format^41^ and the MD simulations were performed by using the GPU modification to PMEMD^45^ from Amber (version 18)^46^. Preparation of the system for the production runs used the following procedures. For sST2, a 5000-step minimization (steps 1-2500 using conjugated gradient followed by 2500 steps steepest decent) was first carried out in which protein atoms were constrained with a 1.0 kcal/mol/Å^2^ force constant with reference to the crystal structure. After minimization, a 25 ps constant volume and constant temperature (NVT) simulation was performed by setting the temperature of the system to 303.15 K while constraining protein atoms with a 1.0 kcal/mol/Å^2^ force constant with reference to the crystal structure using a time step of 1 fs. Another 1 ns constant pressure and constant temperature (NPT) simulation at 303.15 K and P = 1 atm was performed without constraining protein atoms before the production runs were executed using the time step of 2 fs. The SHAKE^47^ algorithm was used to fix bonds involving hydrogen. The PME method^48^ was used to account for long-range electrostatic interaction and the non-bonded cutoff distance was set at 12 Å and the force-based switching set at 10 Å. For ST2, a 5000-step minimization (steps 1–2500 using conjugated gradient followed by 2500 steps steepest decent) was first carried out in which protein atoms were constrained with a 10.0 kcal/mol/Å^2^ force constant and the phosphorus atoms in POPC were constrained with a 2.0 kcal/mol/Å^2^ force constant with reference to the initial structure. Six equilibration steps were adopted. First, a 25 ps NVT simulation was performed by setting the temperature of the system to 303.15 K while constraining protein atoms with a 10.0 kcal/mol/Å^2^ force constant and the phosphorus atoms in POPC with a 2.0 kcal/mol/Å^2^ force constant with reference to the initial structure using a time step of 1 fs. Second, same procedures as the first were used but the constraints to the protein atoms were reduced to a 5.0 kcal/mol/Å^2^ force constant. Third, a 25 ps NPT (T = 303.15 K, P = 1 atm) simulation was performed by constraining protein atoms with a 2.5 kcal/mol/Å^2^ force constant and the phosphorus atoms in POPC with a 1.0 kcal/mol/Å^2^ force constant with reference to the initial structure using a time step of 1 fs. The constant surface tension was set to zero on the xy plane. Forth, same as the third for a 50 ps simulation but the constraints to the protein atoms and the phosphorus atoms in POPC were reduced to 1.0 and 0.5 kcal/mol/Å^2^ respectively. Fifth, same as the forth for a 100 ps simulation at a time step of 2 fs but constraints to the protein atoms and the phosphorus atoms in POPC were reduced to 0.5 and 0.1 kcal/mol/Å^2^ respectively. Sixth, same as the fifth but only the protein atoms were restrained to a 0.1 kcal/mol/Å^2^ force constant. Another 1 ns equilibration at a time step of 2 fs was performed without imposing constraints to the protein atoms before the production run was initiated.

In the aMD simulations, we used the average total and dihedral energy of 2 ns equilibrium run prior to the starting conformation at 10ns of cMD simulation to calculate three threshold potential energies, called aMD1, aMD2 and aMD3, described in our previous work^25^. The conformation at 10 ns of cMD simulations was selected as the starting conformation for conformational sampling in aMD. Because the rate for the starting conformation to be trapped at local minima differed among AMD1–3, we performed 80, 81.5 and 61.1 ns of simulations using the aMD1–3 parameters respectively.

### Principal component analysis (PCA)

The procedures to perform the PCA have been described previously^25^. Briefly, thirteen crystal structures from the eleven protein complexes containing the ectodomain of the IL-1R members (IL-1R1, IL-1R2,IL-1RAcP, ST2, IL-18R1 and IL-18RAcP) were analyzed in the PCA implemented in the Bio3D version 2.3^49^ in the R program 3.6^50^. Among the crystal structures, only Cα atoms (total of 184) coordinates of non-gap amino acids determined in the sequence alignments were used in the PCA to define the backbone conformational space. Conformations obtained from each trajectory from the simulations were then projected to the principal component (PC) space to analyze the conformational changes. In the PCA, the first three PCs contributed 47.17, 30.64 and 10.95% (Table S1) to the overall structural displacements respectively.

### Cluster analysis of sST2 conformations obtained from the aMD1 simulation

We used the template project module from MSMBulider v3.8^51,52^ to perform the analysis. Snapshots of the protein conformations from the aMD1 simulation were taken in every 50 ps. The conformational space of sST2 was defined by the torsional angles of the backbone and the side chains of 9 amino acids (sequence: 201–210, VKDEQGFSL) between the D2 and the D3 domain of ST2. The torsional angles were converted into features that were subjected to time-lagged independent component (tIC) analysis^53–55^ using a lag time of 500 ps. To determine the 15 cluster groups, we performed the K-Medoids clustering analysis^52^ of the loop conformations using the first five tICs representing five slowest relaxation degrees of freedom. The conformations closest to the centroid of each cluster group were used as representative conformations shown in Fig. S4.

### Markov State Model construction

The pyEMMA package 2.5.7^56^ was used in the Markov State Model (MSM) analysis. Snapshots of sST2 or ST2^ECD^ conformations at every 50 ps obtained from the simulations were used in the analysis. Only the backbone dihedral angles of the 9 amino acids at the loop were included in the tIC analysis. We varied the lag time from 0.05 to 20 ns and determined 4, 15, 15 ns were suitable to obtain the plateau values of the implied timescales in the analyses of sST2, ST2^ECD^ and sST2 + ST2^ECD^ ensembles (see Fig. S9). To determine the number of microstates to model the transition events, we varied the number of cluster groups from 10 to 600 and found 300 clusters giving a plateau VAMP-2 score value. In the Robust Perron Cluster Analysis (PCCA+ ) analysis^57^, we varied the number of macrostates from 6 to 12 and found the 12-macrostate model giving good agreement between the estimated and the predicted transition probability in the Chapman-Kilmogorow test (CK test, cf. Figs. S10 and S12)^58^.

## Results

### The D3 domain of sST2 and ST2 exhibited great flexibility observed in the early time of MD simulations

We have previously reported that the D3 domain of ST2^ECD^ (ST2^D3^) underwent a large amplitude rotational motion relative to the D1 and D2 domains (ST2^D1D2^)^25^. Using different force fields parameters including three glycosylated asparagines in sST2, we reached a similar finding in this study (Fig. S1). We first aligned the D1D2 domain of ST2 conformations to the crystal structure of ST2^ECD^ in complex with IL-33 to determine the backbone motions of each domain for the initial 108 ns of sST2, ST2^1-3^ using the cMD simulations. We found the deviation of sST2^D2^ was small whereas that of sST2^D1^ was larger. Changes of the backbone root-mean-squared-deviations (RMSDs) of sST2^D3^ were dramatic and the values rose to around 20 Å at 1.4 ns before increasing to 50 Å at 85 ns. In the case of ST2, we found similar patterns of backbone deviations among the D1-D3 domains. For example, ST2^D2^ remained close to the crystal structure of ST2 and ST2^D1^ deviations were less than that found in sST2^D1^. Increases of backbone RMSDs in ST2^D3^ however varied in three different ST2 simulations. Rises of RMSDs to 40–60 Å all occurred at a later time in ST2 compared with that in sST2. In all four 108 ns simulations of sST2, ST2^1-3^, the average RMSDs for ST2^D1^, ST2^D2^, ST2^D3^ were 3.71 ± 0.48, 2.50 ± 0.55 and 43.09 ± 13.31 Å respectively. The data confirmed that the large interdomain motion between ST2^D1D2^ and ST2^D3^ is a shared property between sST2 and ST2 and is unaffected by the transmembrane and the cytosolic domains of ST2.

The large RMSD values of the D3 domain imply flexible motion of ST2^D3^, but they are not ideal classifiers to distinguish different ST2 conformations. We then employed the PCA to analyze the conformations of sST2 and ST2^ECD^ obtained from the long-time MD simulations following the procedures reported previously^25^. In PCA, the overall backbone motion of the protein was decomposed to different types of collective motions described by PCs^59,60^. The collective motions of the first six PCs were provided in the Supplemental Materials for reference. Only PC1 showed a large amplitude motion of ST2^D3^ relative to ST2^D1D2^. The remaining PC2-PC6 characterized either collective backbone vibration or twisted motions. sST2 and ST2^ECD^ conformations from the simulations projected to the PC space further allowed to analyze the differences of the conformational changes associated with each type of motion^25^.

**Figure 2:**
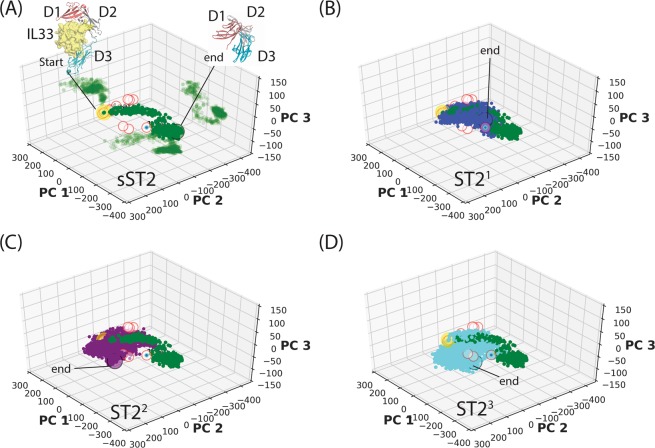
Projections of ST2 conformations to the three-dimensional PC1–3 space. In (**A**), sST2 conformations obtained from the cMD simulations are colored in green circles. The conformations are also projected to the two-dimensional PC1-PC2, PC2-PC3 and PC1-PC3 spaces. The starting conformation is shown in a yellow circle and the end conformation is depicted in a black circle. In (**B-D**), conformations of the ectodomain of ST2^1^, ST2^2^, and ST2^3^ are projected to the PC1–3 space in blue, purple and cyan circles respectively. Crystal structures used to construct the PC space are shown in red open circles. The cyan dot with a red circle corresponds to the antagonist peptide bound IL-1R1 structure (PDBID: 1G0Y). Figures are prepared with the R program 3.6 (www.r-project.org) and Matplotlib 3.1.3 (www.matplotlib.org).

Although the complete description of ST2 conformational motions required all PCs (552 degrees of freedom) in the analysis, we have shown that the first three PCs (PC1–3) provided a reasonable characterization of the large amplitude ST2 motions in plots^25^. For example, the first three PCs accounted for 87% of structural variations in the crystal structures here. After the projections of ST2 conformations to PC1-PC3, we found that conformations of sST2 were mapped to a circular path along PC1 and PC2 (Fig. 2A) suggesting a nonlinear coupled motion of large amplitude domain motions (PC1) and collective backbone vibration (PC2) dominated the conformational transition. In the PC1-PC3 space, we also found that sST2 visited conformations close to the cytokine-bound IL-1R1/2 conformations (red open circles) and was trapped in a region close to the conformation of the antagonist-bound IL-1R1 (PDBID: 1G0Y, the cyan dot in Fig. 2A). The final sST2 conformation in Fig. 2A showed that sST2 adopted a close conformation in which the D1 and D3 domains contacted each other^25^.

In the ST2 conformations obtained from the cMD simulations, circular paths along PC1 and PC2 were not found (Fig. 2B-D). This suggests that ST2^ECD^ underwent less degree of the large amplitude motion along PC1. ST2^ECD^ mainly sampled a larger conformational space along PC2 and PC3, reflecting coupled backbone vibration and twisted motions. In the 386 ns of ST2^1^, 759 ns of ST2^2^, and 1.53 μs of ST2^3^ cMD simulations, conformation overlaps between ST2^1^ and sST2 are greater in the PC1–3 space indicating their similar relaxation paths. The PCA is thus a useful tool to analyze and differentiate the large amplitude conformational transitions between sST2 and ST2. To examine the overall shape changes in ST2, we monitored the distance of the center-of-mass (COM) between the D1 and the D3 domains (COM^D1–D3^) and the radius of gyration (ROG) of the proteins. Fig. S2 showed that COM^D1–D3^ of sST2 decreased quickly in less than 100 ns. For ST2, the D1 and the D3 domains made close contacts at a slower pace in the ST2^1^ and ST2^2^ simulations. In contrast, no contact was established in ST2^3^ over the 1.53 μs simulation. Changes of the ROG values showed a similar trend suggesting that the ectodomain of ST2^3^ relaxed to an extended conformation different from the globular shapes found in sST2, ST2^1^ and ST2^2.^

### Extensive mapping of the sST2 conformational space was facilitated by the aMD simulations

**Figure 3:**
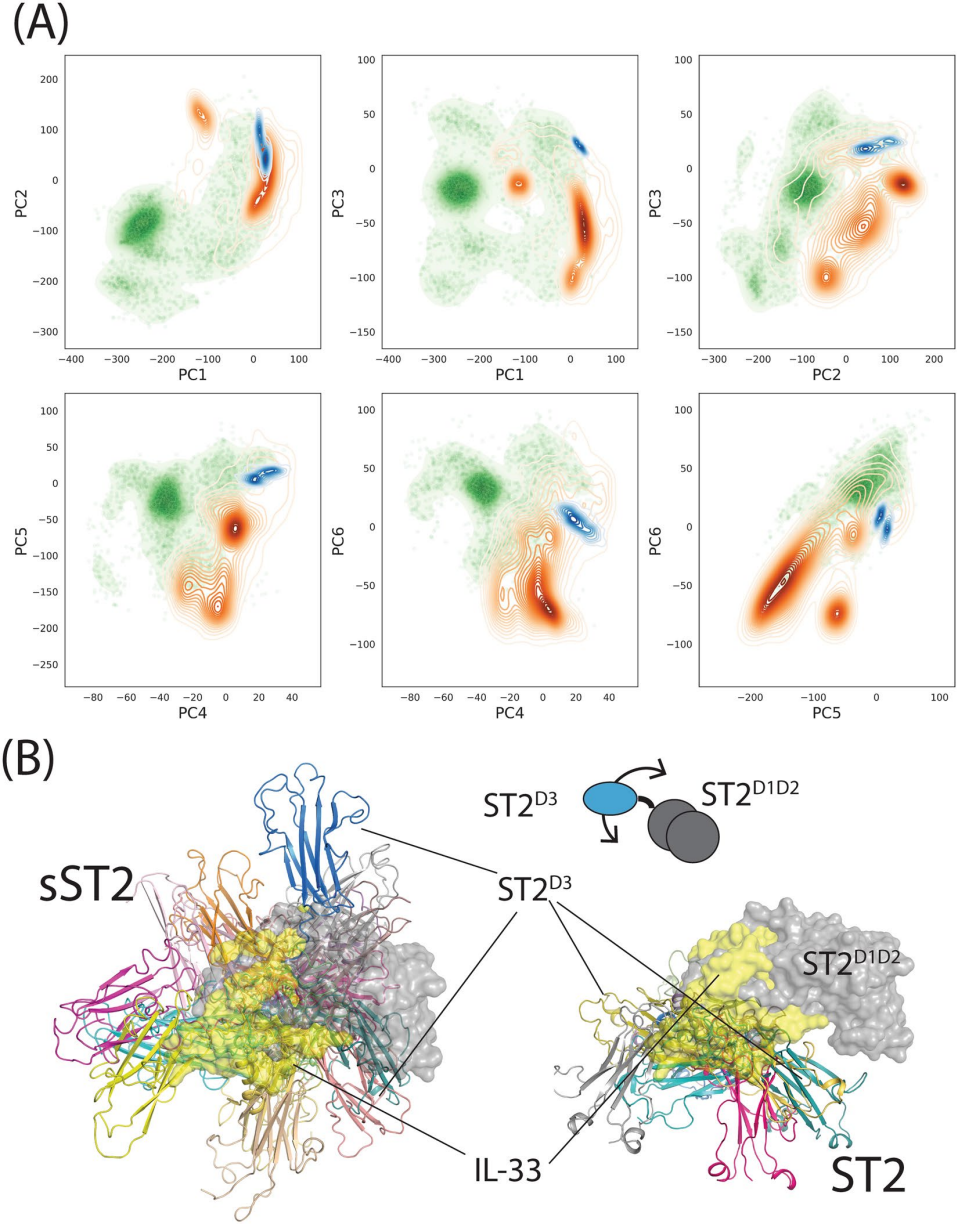
Projection of sST2 and ST2 conformations to PC1-PC6 and representative conformations of sST2 and ST2 from simulations. In (**A**), sST2 (green) and ST2 (orange) conformations are projected to a two-dimensional PC space from PC1 to PC6. Projected conformations of mouse ST2 from the mouse IL-1RAcP/ST2/IL-33 simulation are shown in blue contour maps. In (**B**), selected representative conformations of sST2 and ST2 from the cluster analysis are shown. All conformations are aligned to the crystal structure of ST2^D1D2^ (grey surface) and arranged that the crystal structure of ST2^D1D2^ is facing forward. The crystal structure of IL-33 is displayed as a yellow surface model. Figures are prepared using the R program 3.6 (www.r-project.org), Matplotlib 3.1.3 (www.matplotlib.org) and PyMOL 2.3.0 (www.github.com/schrodinger/pymol-open-source).

We have previously demonstrated that aMD allows efficient conformational sampling of IL-1R1^ECD^ and ST2^ECD^ to cover broad conformational spaces inaccessible to cMD using the same simulation times but different simulation paramters^25,60^. To examine the outcomes of the force field parameters used in this study, we evaluated three sets of acceleration parameters (aMD1–3) in the aMD simulations of sST2 (Fig. S3). Modifications of the potential energy functions by aMD1–3 parameters were expected to sample the sST2 conformations from an elevated to a higher energy states^25^. This was shown by different densely populated regions in PC1–3 accessed by the three aMD simulations (see Fig. S3A–C). Collectively, sST2 conformations obtained from cMD and aMD1–3 (a total of 240 ns simulations) mapped to a larger PC1–3 space than those of ST2^ECD^ from a total of 2.7 μs cMD simulations (cf. Fig. S3D). To further determine the conformational differences between sST2 and ST2^ECD^, we analyzed their projections to the first six PCs that account for 99% of structural changes (or variances) in Fig. 3. We used the conformations of the mouse ST2 from the simulations of the complex of mouse IL-1RAcP/ST2/IL-33 (Fig. 1A) as a reference to represent the IL-33-bound ST2 state. Figure 3A showed that ST2^ECD^ generally sampled a more restricted PC space. The plots also indicated that motions along the PC1, PC2 and PC4 differ between sST2 and ST2^ECD^ because of less overlapping population. Because most of the sST2 conformations were sampled from aMD simulations, the dense populations in the PC space do not correspond directly to the lowest free energy of the unmodified potential surface. To visualize the structural differences between sST2 and ST2^ECD^, we performed the K-Mean cluster analysis using the first seven PCs (99% of variances) and identified 22 and 13 cluster groups in the sST2 and ST2^ECD^ conformations respectively. Representative conformations closest to the centroid of each cluster group were aligned to the crystal structure of ST2^D1D2^ for comparison. Figure 3B highlighted that the D3 domain of sST2 indeed underwent greater rotational and twisted motions than that of ST2^ECD^.

### Performing multi-trajectory equilibrium cMD simulations using diverse sST2 conformations to investigate the interdomain loop dynamics

Although aMD efficiently sampled the sST2 conformations at a shorter simulation time, estimation of the conformational transition kinetics requires trajectories governed by unmodified potential energy functions. To study the global conformational transition, cMD simulations of sST2 started with diverse conformations in the free energy surface are needed. Our analysis showed conformations sampled by the aMD1 simulation represented the smallest modified potential energy function yet covered a large PC1–3 space (Fig. S3A). We also reported that flexibility of the loop between ST2^D2^ and ST2^D3^ led to diverse sST2 conformations^25^. To select sST2 conformations for multi-trajectory simulations, we used the torsional angles of the backbone and side chains of 9 amino acids in the loop region (sequence: 201–210, VKDEQGFSL) to represent sST2 conformations obtained from the aMD1 simulation. The time-lagged independent component (tIC) analysis of the torsional angles was used to determine the two slowest degrees of freedom of the loop motion (tIC1–2). After mapping the sST2 conformations to tIC1 and tIC2, we performed the K-Medoids cluster analysis^52^ to identify 15 cluster groups distributed in five distinctive regions (Fig. S4). Representative conformations in each cluster group indeed showed a wide range of sST2^D3^ orientations relative to sST2^D1D2^ (Fig. S4). These 15 conformations were used as the initial conformations for 300 ns cMD simulations to generate an equilibrium ensemble of sST2. By monitoring the values of COM^D1–D3^ in the 15 cMD simulations, we found that sST2 either collapsed to conformations in which ST2^D1^ contacted ST2^D3^ or adopted extended conformations at the end of the simulations (Fig. S5).

**Figure 4:**
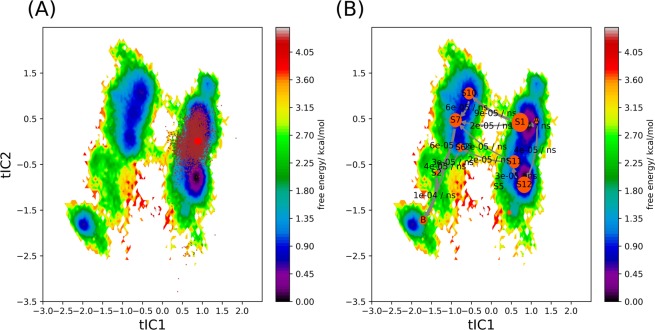
The weighted free energy surface constructed from the loop conformations of sST2 obtained from cMD simulations and the conformational transition flux of sST2 based on the MSM. In (**A**), conformations of sST2 relaxed from the IL-33 bound ST2 structures (101 ns, brown dots) are mapped to the weighted free energy surface of sST2 built from the loop conformations of a total of 4.5 μs of cMD simulations. The ST2 crystal structure is shown in a red dot. In (**B**), the transition flux from the right most macrostate (denoted as A) to the left most macrostate (denoted as B) along the slowest degree of freedom (tIC1) based on the MSM is displayed. The transition directions and rates between different macrostates are labeled. Figures are prepared with pyEMMA 2.5.7 (www.emma-project.org).

To analyze the conformational transition kinetics of ST2, we employed Markov State Model (MSM) implemented in pyEMMA^56^. In this analysis, only the backbone torsional angles of the loop were used as the reaction coordinate to represent the conformational transition. We have varied the lag time and numbers of cluster groups to determine parameters suitable for the MSM. We found 80 steps (or 4 ns lag time) and 300 cluster groups gave converged stationary distribution and weighted free energy surface in tIC1 and tIC2 (Figs. S6, S7). Based on the analysis, sST2 conformations were divided into two connected valleys on the free energy surface (Figs. S6, 7). The stationary distribution indicated a larger population of sST2 at the right valley with a corresponding lower free energy of the loop segment (tIC1 = 1, Fig. S7). The trajectory started from the IL-33 bound sST2 in the cMD simulation also mapped to the right valley (Fig. 4A). The conformational transition flux corresponding to the slowest implied timescale (~5728 ns, Fig. S8) displayed a population flow (or conformational changes) from the lower left valley (tIC1 = −2, tIC2 = −2, Fig. S9) to the right valley. To depict the conformational transition flow using a smaller number of metastable states, we performed the Robust Perron Cluster Analysis (PCCA+ ) analysis^57^. After conducting the Chapman-Kilmogorow test (CK test)^58^, we found that 12 metastable states were required to give agreement between the predicted and estimated metastable state relaxation (Fig. S10). By tracking the flux from the right-most metastable state to the left-most state along tIC1 (the slowest degree of freedom of the loop motion), we found that sST2 conformations on the right valley channeled via two pathways (from S_1_ to S_10_/S_7_ and S_11_ to S_7_/S_6_ respectively) to the left valley before reaching B (S_8_) (Fig. 4B). As demonstrated, the broad conformational space of sST2 generated from the cMD simulations provided a necessary free energy landscape coverage to analyze the ST2^ECD^ conformations obtained from the cMD simulations.

### The conformational transition kinetic of ST2^ECD^ in ST2 and its relation to sST2

**Figure 5:**
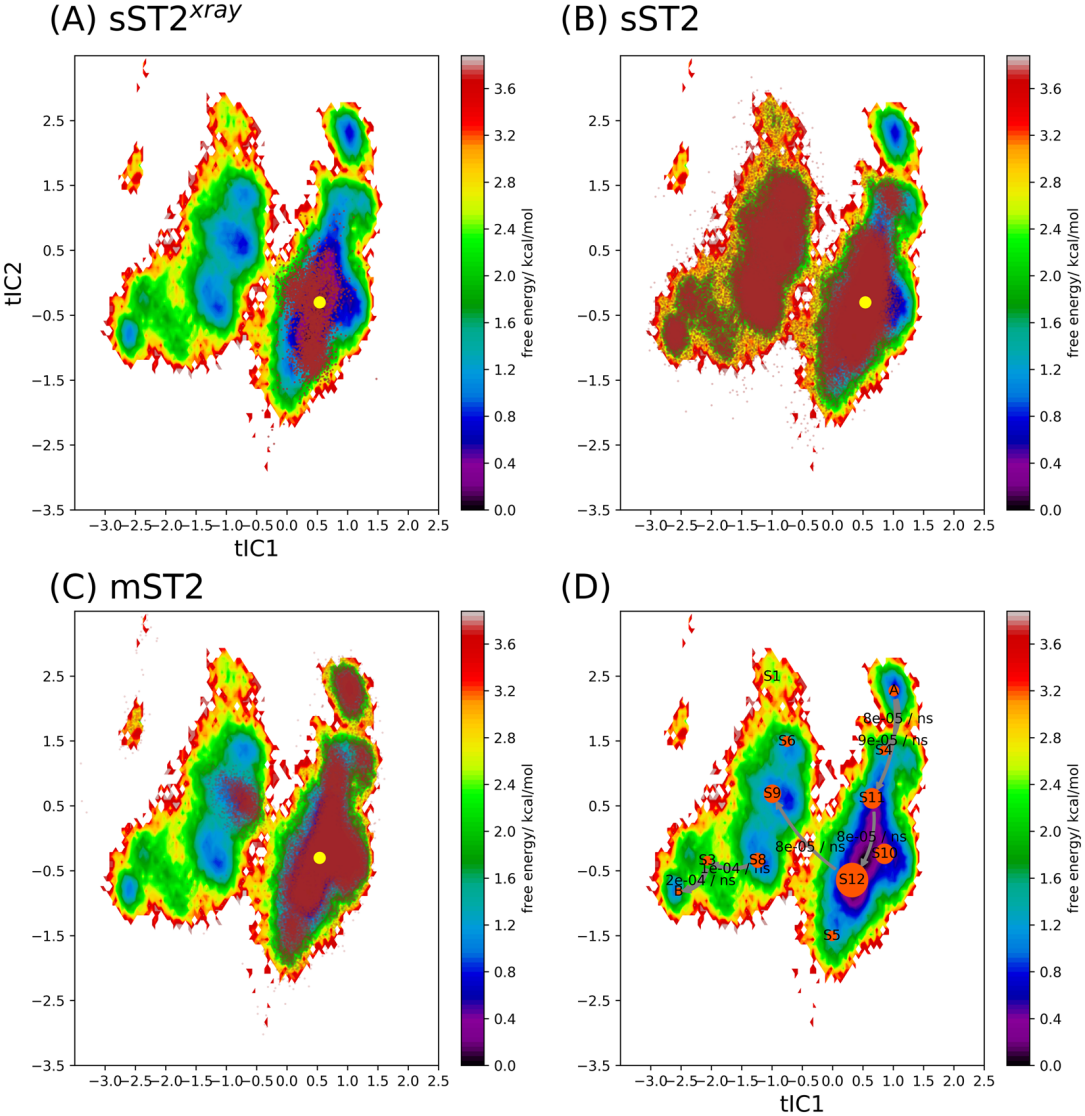
Projection of sST2 and ST2 conformations to the weighted free energy surface based on sST2 and ST2. Conformations of sST2 started with sST2^xray^ are shown in (**A**). Conformations of sST2 and ST2 are shown in (**B**) and (**C**). In (**D**), dominant fluxes (>2 * 10^−5^/ns) flowing from the right most macrostate (denoted as A) to the left most macrostate (denoted as B) along the slowest degree of freedom (tIC1) based on the MSM are displayed. Figures are prepared with pyEMMA 2.5.7 (www.emma-project.org).

**Figure 6:**
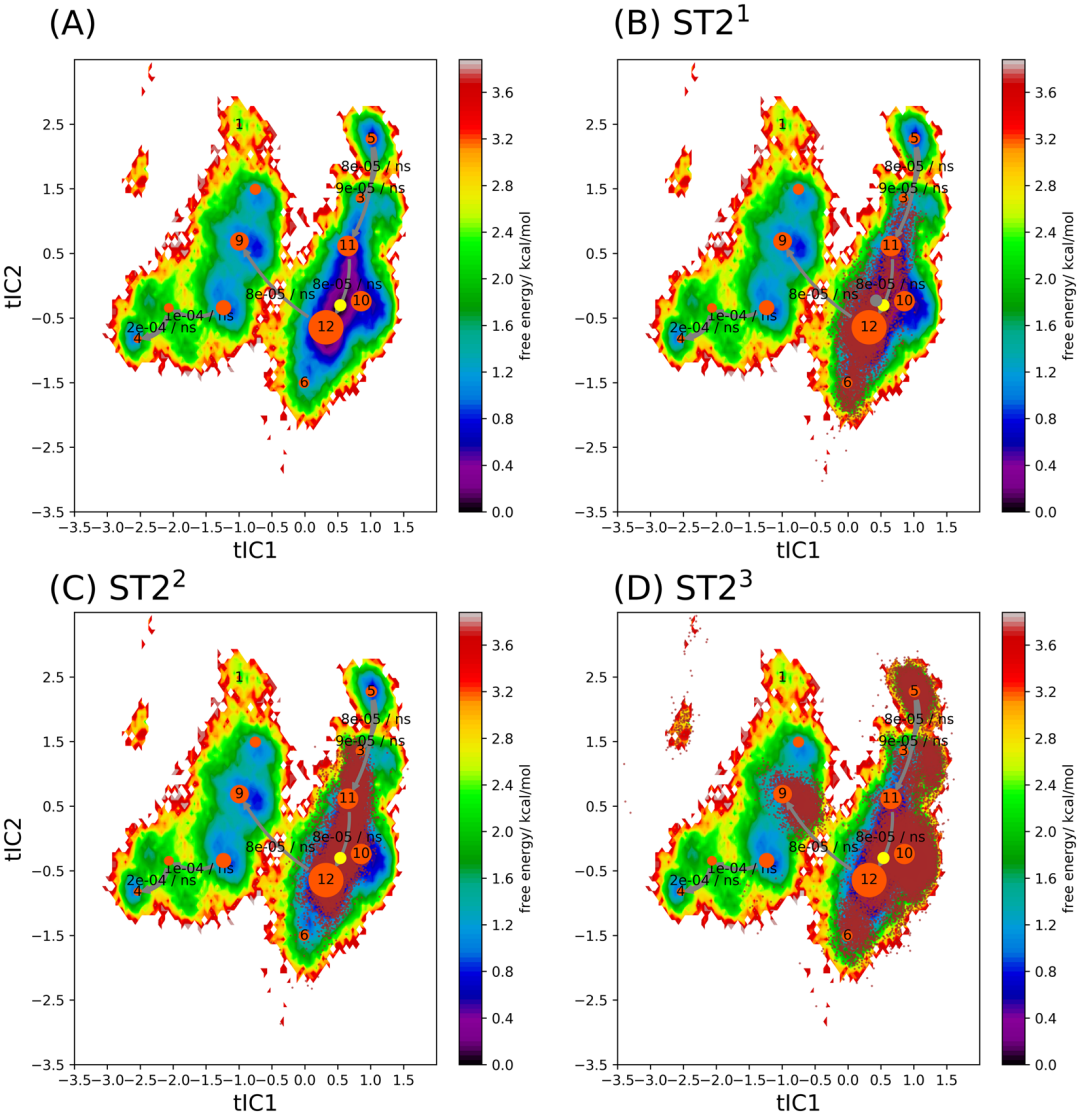
Dominant fluxes of the conformational transitions in sST2 + ST2^ECD^ based on the MSM and projection of ST2 conformations to the weighted free energy surface. In (**A**), the dominant fluxes flowing from the right most macrostate (denoted as 5) to the left most macrostate (denoted as 4) along the slowest degree of freedom (tIC1) based on the MSM are displayed. In (**B**–**D**), conformations obtained from the ST2^1^, ST2^2^ and ST2^3^ simulations are projected to the weighted free energy surface respectively. The transition directions and rates between different macrostates are labeled. The ST2 crystal structure is shown in a yellow dot. Figures are prepared with pyEMMA 2.5.7 (www.emma-project.org).

To analyze conformational transition of ST2 alone, we used the same parameters and procedures in MSM as those in sST2. Fig. S11 showed that ST2^ECD^ conformations were mapped to three regions. The slowest timescale of the conformational transition for ST2 was estimated at ~299 ns (Fig. S8). The first 307 residues (sequence 19–323) of ST2 (i.e. ST2^ECD^) and sST2 are identical. To compare sST2 with ST2^ECD^ using the same tICs, we combined the loop conformations of sST2 and ST2^ECD^ in the following analysis. Figure 5A showed all conformations were divided to 12 metastable states (macrostates) and the IL-33 bound ST2 (ST2^xray^) was located in S_10_. Although the overall free energy landscape of sST2 + ST2^ECD^ was modified (cf. Figs. 4A and 5A, tIC2 changes sign in the free energy surface between sST2 and sST2 + ST2^ECD^), yet the free energy surface was dominated by sST2 conformations (Fig. 5B). Figure 5C further indicated that ST2^ECD^ sampled additional loop conformations not visited by sST2 after 1 μs of simulations. Similar to the findings in the sST2 analyses (Fig. 4B), the major population flux along tIC1 between two valleys flowed from S_12_ to S_9_ (S_1_ to S_10_ in Fig. 4A) but much less via the second pathway before reaching state B (Fig. 5D). The second major flux (S8 → S3 → B) is associated with sST2 not ST2^ECD^. Among the ST2 conformations, we found that conformations of ST2^1^ and ST2^2^ were localized at the right valley (Fig. 6B-C). These conformations corresponded to the relaxation of ST2^ECD^ to a final state that ST2^D1D2^ made close contact with ST2^D3^ (Fig. S2). In contrast, conformations obtained from ST2^3^ were mapped to three different regions including those in S_9_ not accessed by ST2^1^ or ST2^2^ (Fig. 6D).

**Figure 7:**
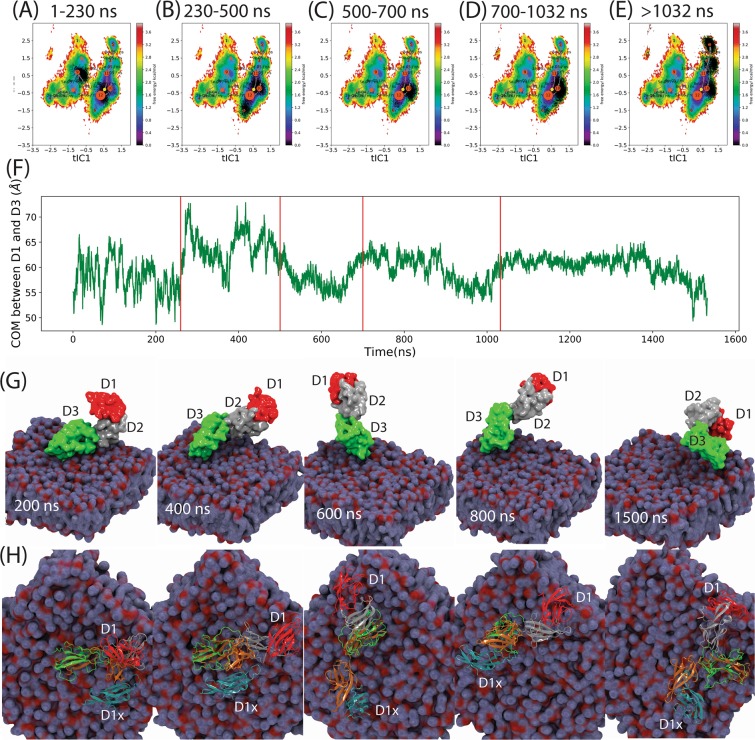
Time evolution of the conformations obtained from the ST2^3^ simulation. In (**A**–**E**), ST2 conformations (black dots) are projected to the weighted free energy surface in the time window of 1–260, 260–500, 500–700, 700–1032 and >1032 ns. In (**F**), the times at 260, 500, 700, and 1032 ns are drawn as red vertical lines in the COM^D1–D3^ plot. In (**G**), snapshots of ST2 conformations at 200, 400, 600, 800 and 1500 ns from the ST2^3^ simulation are shown. The D1, D2, and D3 domains are displayed as the surface representation and colored in red, grey and green respectively. The lipid is shown as a quick surface representation. In (**H**), the same ST2 conformations as those in (**F**) are shown in the cartoon representation. The ST2 crystal structure is aligned to the D3 domain of each ST2 conformation and the D1 domain of the ST2 crystal structure (denoted as D1x) is colored in cyan color. The D2 and D3 domains of ST2^xray^ are colored in brown color and the top view of the lipid is used. Figures are prepared using pyEMMA 2.5.7 (www.emma-project.org), Matplotlib 3.1.3 (www.matplotlib.org), and VMD 1.9.4 (www.github.com/schrodinger/pymol-open-source)^27,28^.

Because the loop conformations obtained from ST2^3^ covered a broader range in the free energy surface than ST2^1^ and ST2^2^, we further analyzed the time evolution of the conformations in ST2^3^. In Fig. 7, we divided the trajectory of the ST2^3^ simulation into five different time intervals (1–260, 260–500, 500–700, 700–1032, >1032 ns) guided by the changes of COM^D1–D3^. Figure 7A showed that ST2^ECD^ transited among S_9_, S_10_ and S_12_ in the initial 260 ns. In 260–500 ns (Fig. 7B), ST2^ECD^ underwent conformational transition by shuttling among S_12_, S_11_ and S_10_. This paralleled the large variations of COM^D1–D3^ in Fig. 7F. In 500–700 and 700–1032 ns, ST2^ECD^ relaxed to the conformational state localized densely in S_11_ and S_10_ (cf. Figures 7C,D and 5). In >1032 ns, ST2^ECD^ underwent smaller amplitude interdomain rotation and reached other local regions (S_3_, S_5_) not sampled by sST2 (Fig. 7E). In Fig. 7G, we showed the arrangement of the D1-D3 domain in ST2 at 200, 400, 600, 800, and 1500 ns corresponding to five time-windows. We also aligned ST2^xray^ to ST2^D3^ to demonstrate the rotation of ST2^D1D2^ from the IL-33 bound ST2^xray^ structure in Fig. 7H. Figure 7H demonstrated that the ST2^ECD^ conformation was close to ST2^xray^ at 200 ns indicated by the close proximity between the D1 and the D1x (D1 of ST2^xray^) domains. After 260 ns, ST2^D1D2^ underwent significant rotation relative to ST2^D3^ and transitioned to extended conformations giving large COM^D1–D3^ values at ~70 Å in Fig. 7F. In 500–700 ns, ST2^ECD^ adopted a conformation that the D1 domain was about 180° from the D1x domain in ST2^xray^. After 700 ns, ST2^ECD^ relaxed to the local free energy minima and assumed extended conformations until 1500 ns.

### **Salt-bridge interactions contribute to the stabilization of the ST2**^**ECD**^**conformations after long time MD simulations**

**Figure 8:**
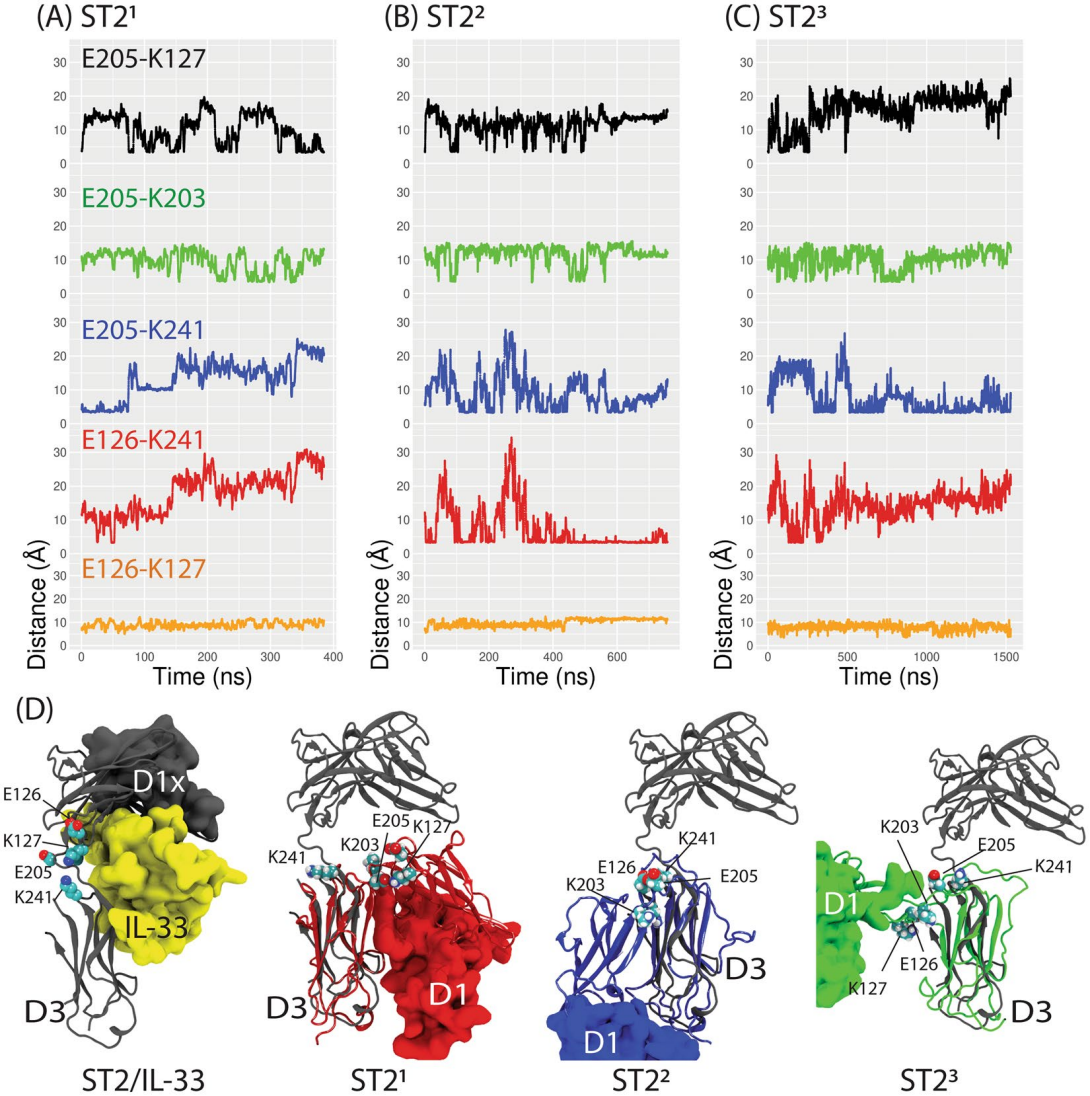
Distances between pairs of salt-bridge interactions and snapshots of ST2^ECD^ conformations from the ST2 simulation. In (**A**–**C**), distances between the acidic group of Glu and amine group of Lys in the pairs of E126-K241 (red), E205-K127 (black), E126-K127 (orange), E205-K203 (green), and E205-K241 (blue) from the ST2^1^, ST2^2^, and ST2^3^ simulations are shown. In (**D**), the crystal structure of ST2/IL-33, the final snapshots of ST2 from the ST2^1^, ST2^2^, ST2^3^ simulations are shown in grey, red, blue, and green colors respectively. The D3 domain of ST2^xray^ is aligned to ST2^ECD^ and IL-33 is colored in yellow. Amino acids participating in salt-bridge interactions are shown in a ball-and-stick model and labeled. Carbon, oxygen, nitrogen and hydrogen atoms are colored in cyan, red, blue and white. Figures are prepared using Matplotlib 3.1.3 (www.matplotlib.org) and VMD 1.9.4 (www.ks.uiuc.edu/Research/vmd)^27,28^.

Based on the COM^D1–D3^ values, ST2 conformations started with three different initial conformations relaxed and stabilized to different conformations. We asked if specific interactions contributed to the stabilization of the loop conformations by analyzing the final snapshots of each ST2 simulation. We found several pairs of salt-bridge interactions in the loop region may play a role. They included E126-K241, K127-E205, E126-K127, K203-E205 and E205-K241. We then monitored the distances between these salt-bridge interactions in each ST2 simulation (Fig. 8). We found that K127-E205 occasionally formed hydrogen bonds at the end of ST2^1^ simulation whereas a strong E126-K241 interaction and a weaker E205-K241 interaction were found at the end of ST2^2^ simulation. In the ST2^3^ simulation, E205 and K241 formed a strong salt-bridge interaction at the end of the simulation. These salt-bridge interactions were not observed in ST2^xray^ bound with IL-33 and likely contributed to the stabilization of different ligand-free ST2 conformations.

## Discussions

Attenuation of cytokine mediated cellular responses by soluble cytokine receptors can limit the cytokine pool in circulation^18^. Several mechanisms of soluble cytokine receptors production have been reported^7^ including sST2 that is produced via alternative splicing^61^ before excretion to extracellular milieu. Aberrant production of sST2 is associated with severity in graft versus host disease^62–68^, cardiac transplantation rejection, IBD and other chronic diseases^69–71^. Further, persistent activation of ST2/IL-33 axis also contributes to chronic type 2 inflammation in asthma^17^ and atopic dermatitis^72,73^. Despite the different role of membrane-bound and soluble cytokine receptors in diseases, our understanding of the differences between them beyond their sequences remains limited. Characterization of differential conformational preferences and dynamics between the soluble and membrane-bound cytokine receptors may assist in developing novel therapeutic approaches to target specific isoforms.

In this study, we found that the IL-33 bound sST2 relaxed to a conformation similar to our previous report^25^. From the microsecond cMD simulations of ST2 using three different orientations of ST2^ECD^, we showed that ST2^ECD^ relaxed to a close state (indicated by small COM^D1–D3^ values) at a slower rate than that of sST2 (300 versus 50 ns). PCA indicated that the close state of sST2 differs from ST2^ECD^ observed at the end of the ST2^1^ and ST2^2^ simulations. In the ST2^3^ simulation where ST2^ECD^ was initially positioned close to the membrane, ST2^ECD^ did not relaxed to a close state but to an extended conformation in 1.5 μs of simulations. Because the IL-33 bound ST2 structure was used as the initial conformation in sST2 and ST2^ECD^, the restrain of ST2^ECD^ by the transmembrane and the cytosolic domains or the interaction of ST2^ECD^ with lipids may influence the conformational relaxation of ST2^ECD^. We also found that the interdomain rotational motion in sST2 causes sST2 relaxed to a local state at <100 ns timescale. In contrast, ST2^ECD^ can relax either to different close states or assume an extended conformation for ~500 ns. Encounter of ST2^ECD^ with the lipids potentially induces a perturbation to modify the free energy surface of ST2^ECD^. Comparison of the representative sST2 and ST2^ECD^ conformations from the aMD and cMD simulations demonstrated that sST2^D3^ may undergo a wider rotational motion relative to sST2^D1D2^ than those in ST2^ECD^. The data were supported by PCA that showed larger negative values of PC1, reflecting a rotational motion of ST2^D3^ relative to ST2^D1D2^, in the sST2 conformations.

Diverse sST2 conformations generated by the aMD simulations also allowed us to perform multi-trajectory cMD simulations to study the kinetics of global conformational transition in sST2. From the 300 ns cMD simulations started with 15 different conformations, we collected conformational transition events of sST2 encompassing different regions in the free energy surface. In comparison, conformational transition events of ST2^ECD^ were generated from microsecond cMD simulations of ST2 using three different orientations relative to the membrane. Because the interdomain loop between ST2^D1D2^ and ST2^D3^ governs their relative orientation, we used the backbone torsional angles of the loop to characterize ST2 conformations and analyzed the kinetics of conformational transition. Using the two slowest degrees of freedom (or tIC), we showed that ST2^ECD^ accessed primarily the lowest free energy surface of sST2 during the simulations. Transition of ST2^ECD^ to another higher free energy valley was transiently found in the ST2^3^ simulation in which ST2^ECD^ interacts with the membrane initially. Combined use of multiple shorter time trajectories connecting free energy valleys have been reported to sufficiently model the transition events in the MSM^74–76^. Our CK tests also confirmed that the Markovian properties of transition kinetics underlies the loop dynamics of sST2. Based on the MSM analysis, the slowest implied timescale of conformational transition in sST2 is estimated at 5728 ns, longer than the 300 ns simulation of sST2 in each trajectory, that sST2 accesses higher energy states. In contrast, the simulation times of ST2 far exceeded the slowest implied timescale of 299 ns for ST2. The timescale should be a reasonable estimate of the conformational transition of ST2 in the subspace visited during the simulations.

Although dihedral angles have been used to identify biomolecular conformations by Schütte’s group employing hidden Markov models^77^, our choice of the dihedral angles of the loop characterizes the conformational transition associated with the relative motion between ST2^D1D2^ and ST2^D3^. Other choices of reaction coordinates^78–80^, such as the principal components and/or interdomain salt-bridge interactions, need to be investigated to determine the global conformational transition rate, ideally with experimental data^81^. Further, our microsecond simulations of ST2 only cover one valley of the free energy surface. Use of diverse initial ST2^ECD^ conformations, similar to that adopted in the sST2 simulations, and a much longer simulation times will be needed to map out the complete ST2^ECD^ conformational space. More extensive sST2 simulations are also needed to obtain accurate estimates of the timescale of the interdomain loop motion and assess if ST2^ECD^ and sST2 exhibit different preferentially relaxed conformations shown in Fig. 6. We anticipate that this computational analysis can be employed to study the conformational flexibility of the membrane-bound IL-1R1 or other cytokine receptors and shed lights on the mechanism of cytokine recruitment and mechanism of signaling^5^.

Relaxation of the IL-33 bound ST2 structure tends to adopt a close ST2 conformation that ST2^D1^ contacts ST2^D3^. This was observed in sST2, ST2^1^ and ST2^2^ using the cMD simulations. We found that salt-bridge interactions around the loop region may assist in the formation of the close ST2 conformations that were subsequently stabilized by the interaction between ST2^D1^ and ST2^D3^_._ Besides the close conformation, the salt-bridge interaction also participates in supporting a metastable extended ST2^ECD^ conformation observed in the ST2^3^ simulation. Modulation of the receptor conformation by interdomain interaction has recently been demonstrated by the study of Li *et al*.^82^. Q268 and Q328 in subdomain 3 and 4 in type I interferon receptor (IFNAR1) was hypothesized to be in close location because of conformational flexibility based on modeling. Experimentally, conformational motion of IFNAR1 bringing Q268C and Q328C in close proximity to enable the disulfide bond formation and lock the conformation was observed.

Recent development of monoclonal antibodies targeting the D1 or the D3 domain of ST2 provides one example that epitopes on different domains in ST2 can be exploited^22^. We did not investigate the differences of the exposed surface areas between sST2 and ST2^ECD^ based on their conformations. The analysis may characterize differentially solvent-exposed epitopes between sST2 or ST2 for antibody development. Our previous study also suggested that targeting transient small molecule binding sites in sST2 conformations may be another strategy to consider^25^. Whether the metastable ST2 conformations mediated by salt-bridge interaction can be further stabilized by small-molecule modulators for therapeutic purpose^60^ remains to be determined.

In summary, we have employed computational simulations of sST2 and ST2 to demonstrate relatively restrained interdomain rotational motion in ST2 in comparison to sST2. This is reflected in the timescale estimates of their conformational transition kinetics. The interdomain salt-bridge interactions were found to contribute to the stabilization of the interdomain loop conformations. Future investigation in the cooperativity between ST2^ECD^ and the TIR domain should shed lights on the recruitment mechanism of IL-1RAcP by ST2/IL33 via the TIR domains.

## Supplementary infomation

Supplementary information.
Supplementary movies.
