# Supplementary material for: Comparative Analyses of the Conformational Dynamics Between the Soluble and Membrane-Bound Cytokine Receptors
Source: Sci Rep. 2020 May 4;10:7399. doi: 10.1038/s41598-020-64034-z (PMC7198498; doi:10.1038/s41598-020-64034-z)
Supplement: Supplementary file 1 — Supplementary information. [file 41598_2020_64034_MOESM1_ESM.pdf]

Comparative Analyses of the Conformational Dynamics Between the Soluble and Membrane-Bound Cytokine Receptors

Chao-Yie Yang

Department of Pharmaceutical Sciences, College of Pharmacy, University Tennessee Health Science Center, Memphis, Tennessee, United States of America

KEYWORDS. Interleukin 1 family, Interleukin 1 receptor type 1, Interleukin 1 receptor like 1, ST2, IL-33, soluble cytokine receptor, Interdomain motion, Molecular dynamics simulation, Principle component analysis, Markov state model, Conformational transition, Kinetic flux analysis.

SUPPLEMENTARY MATERIALS

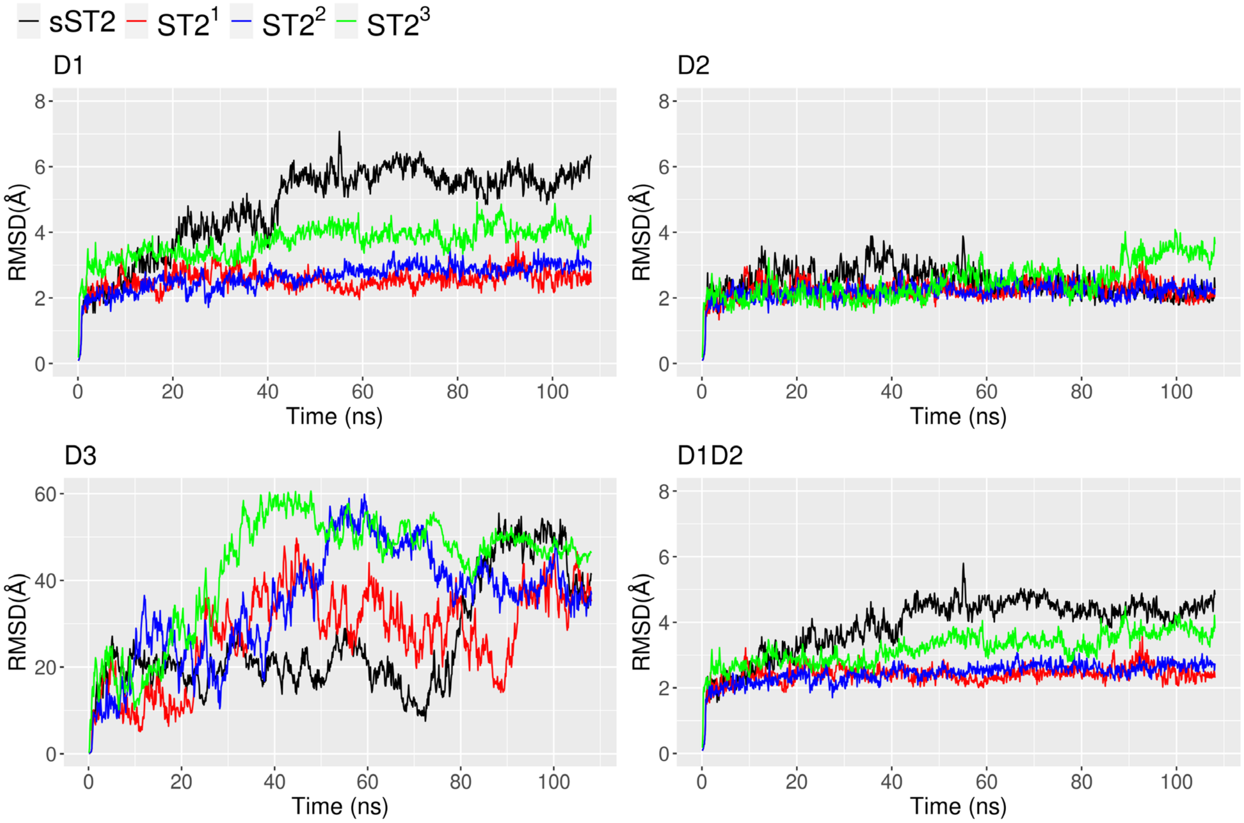

Figure S1. Deviations of the backbone conformations from the IL-33 bound ST2^{xray} in sST2 and ST2 from the initial 108 ns of cMD simulations. For the D3 domain, the ST2 conformations are aligned to the D1 and D2 domain of the ST2 crystal structure before calculating the RMSD of the D3 domain. Figures are prepared with the R program 3.6 (www.r-project.org) and Matplotlib 3.1.3 (www.matplotlib.org).

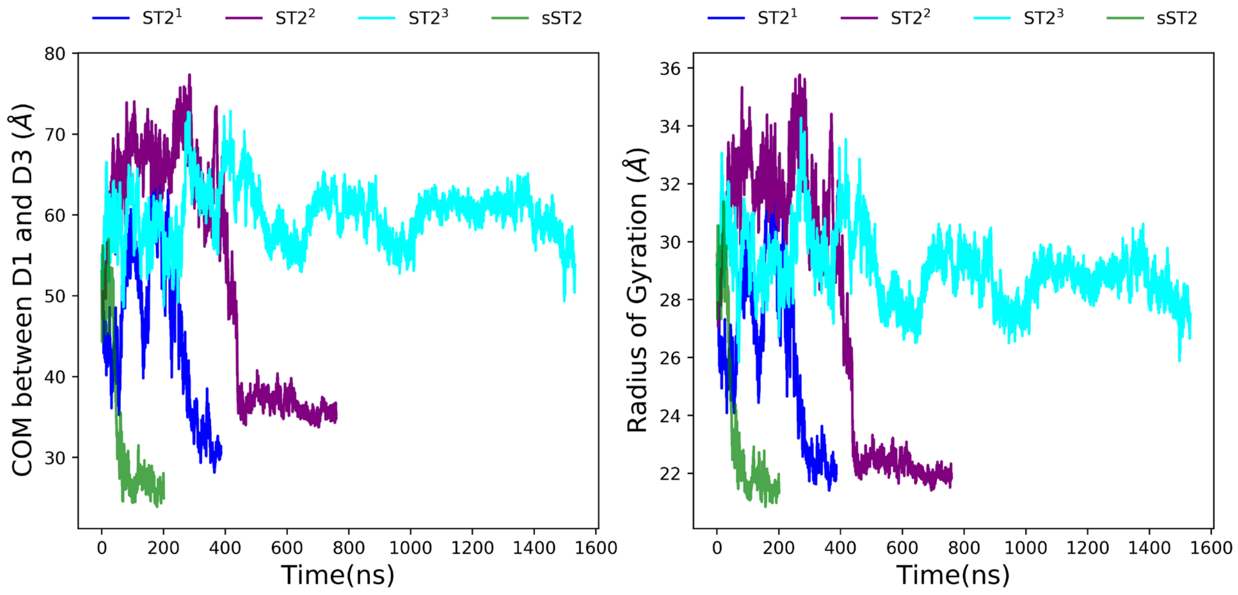

Figure S2. The center-of-mass (COM) between the D1 and D3 domains and the radius of gyration of $ST2^{ECD}$ in the sST2, $ST2^1$, $ST2^2$, $ST2^3$ simulations. Figures are prepared with the R program 3.6 (www.r-project.org) and Matplotlib 3.1.3 (www.matplotlib.org).

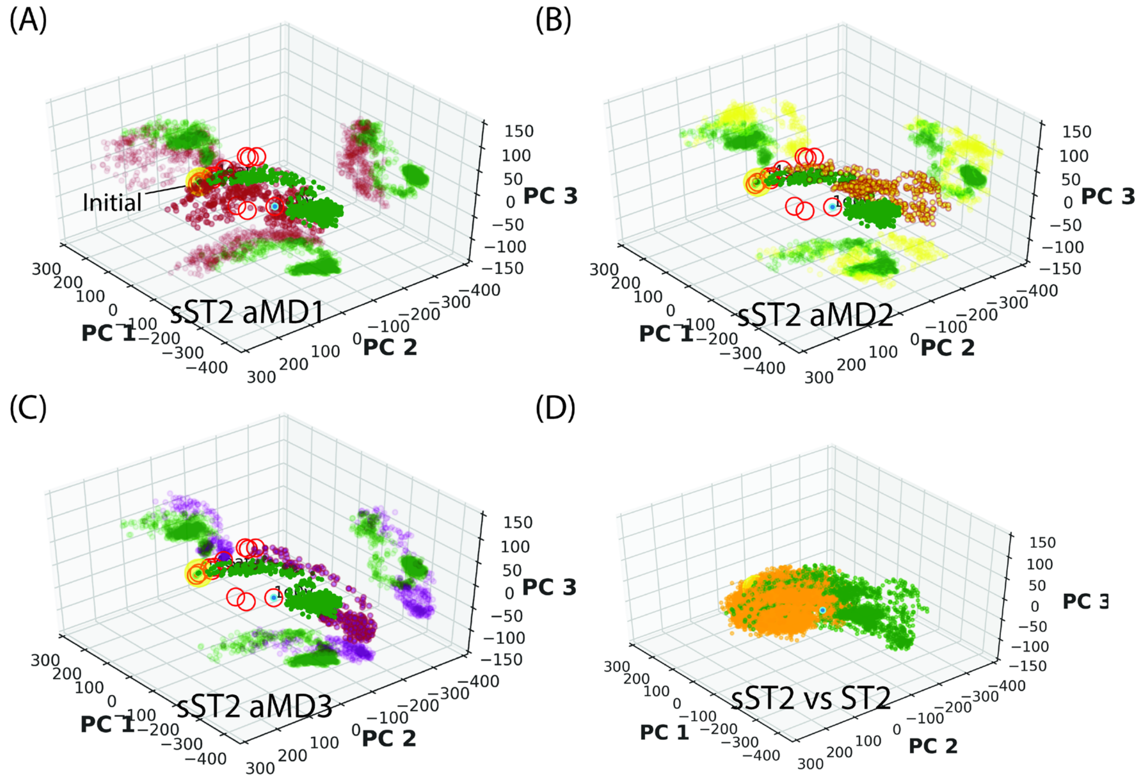

Figure S3. Projections of sST2 conformations from the aMD simulations and the ST2 conformations from the cMD simulations to PC1-PC3 space. In (A-C), sST2 conformations obtained from the aMD simulations using aMD1-3 parameters are shown in brown, yellow, and purple colors. The sST2 conformations from the 108 ns of cMD simulations are shown in green. In (D), all sST2 and ST2 conformations from simulations are shown in orange and green colors. The $ST2^{xray}$ is depicted as a cyan dot in a yellow circle. The antagonist-bound IL-1R1 is shown as a cyan dot in a red circle. Other crystal structures used to construct the PCA are shown in red open circles. Figures are prepared with the R program 3.6 (www.r-project.org) and Matplotlib 3.1.3 (www.matplotlib.org).

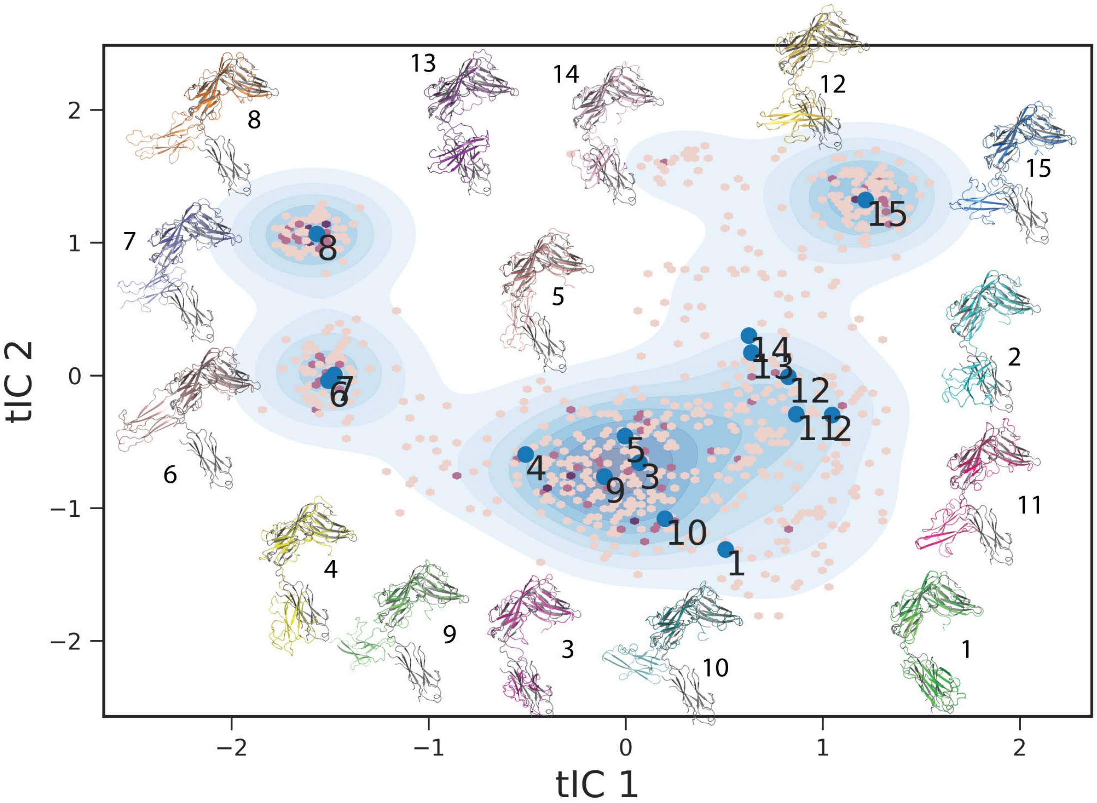

Figure S4. Fifteen cluster groups of the sST2 conformations from the aMD1 simulation determined by the time-lagged independent component analysis using the dihedral angles of the loop conformations. The light orange and purple dots represent each conformation and the blue dots correspond to the centroid of each cluster group. Conformations at the centroid of each cluster group are shown and $ST2^{xray}$ (grey) is aligned to the D1 and D2 domain of each sST2 conformation. These 15 conformations are used as the initial conformations for the 240 ns cMD simulations to sample the global conformational space of sST2.

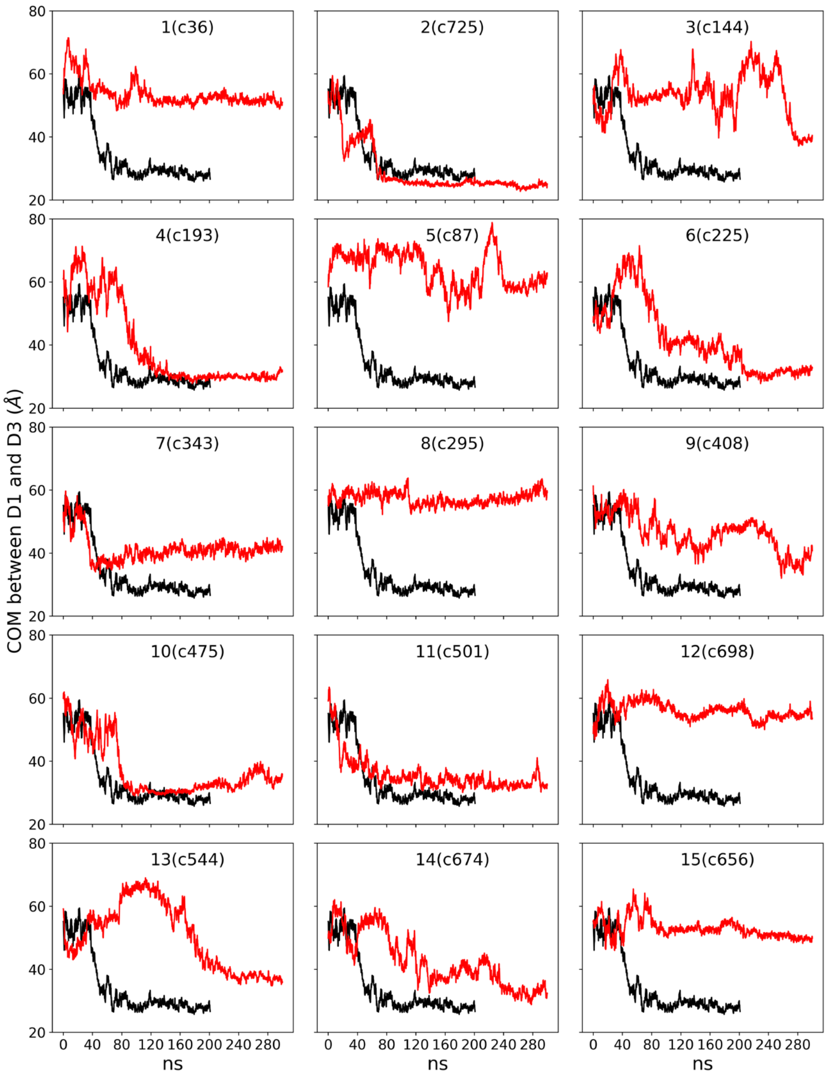

Figure S5. The COM$^{D1\text{-}D3}$ of the sST2 conformations from the cMD simulations started with 15 diverse conformations. Numbers in the parentheses are index numbers of the sST2 conformations from the aMD1 simulation. The black line corresponds to the 108 ns cMD simulation using the ST2^{xray}. Figures are prepared with the R program 3.6 (www.r-project.org) and Matplotlib 3.1.3 (www.matplotlib.org).

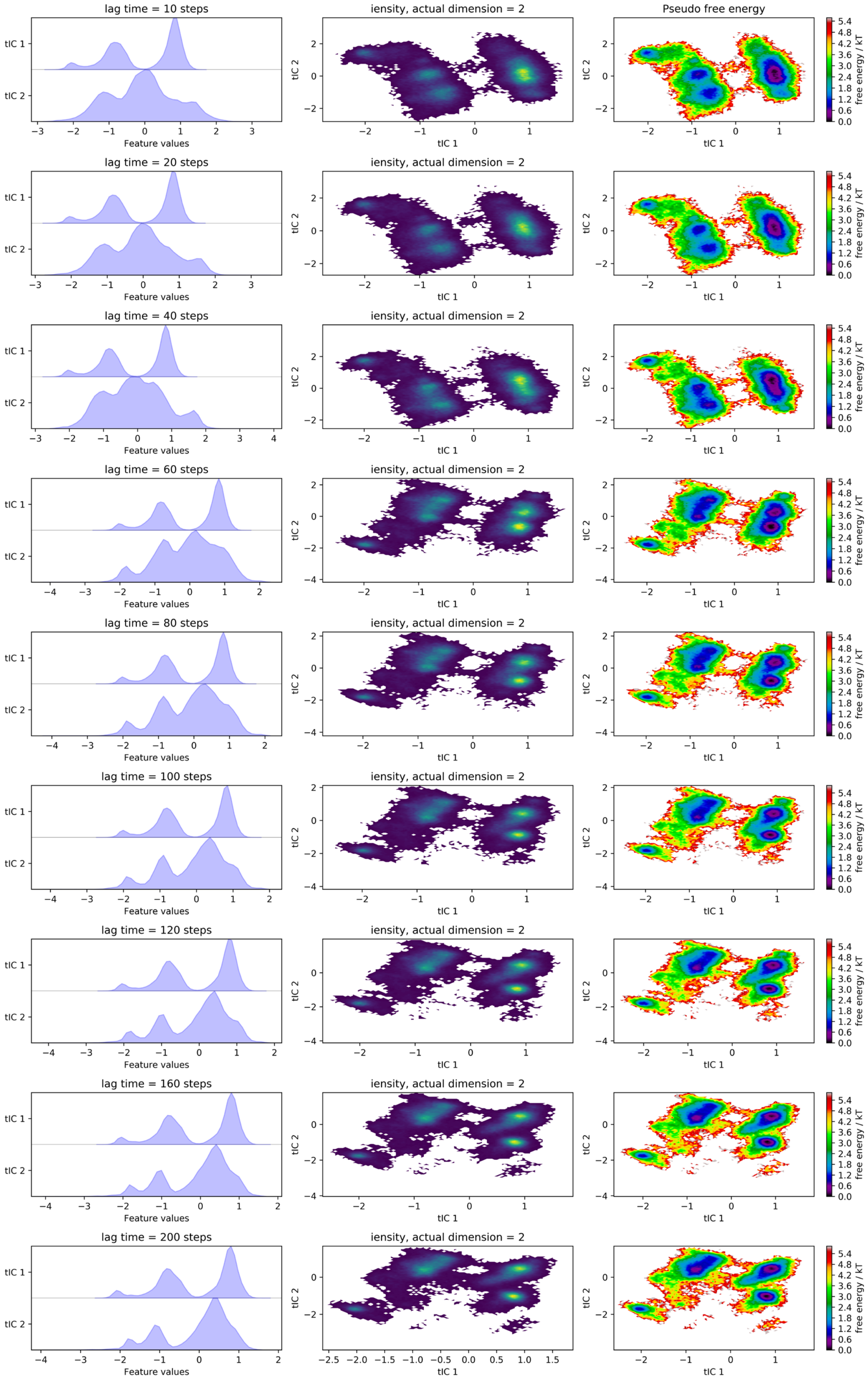
lag time = 10 steps
iensity, actual dimension = 2
Pseudo free energy
lag time = 20 steps
lag time = 40 steps
lag time = 60 steps
lag time = 80 steps
lag time = 100 steps
lag time = 120 steps
lag time = 160 steps
lag time = 200 steps
tIC 1
tIC 2
Feature values
free energy / kT

Figure S6. Variations of the lag time to determine the ideal lag time for the MSM analysis using the sST2 conformations. The lag time is changed from 10 to 200 steps and each step is 50 ps. The left figures are the distributions of tIC1 and tIC2 values. The middle figures are the stationary distribution of sST2 conformations in tIC1 and tIC2. The right figures are the corresponding free energy surfaces. The backbone dihedral angles of the loop between the D2 and D3 domains in sST2 are used as the reaction coordinate in this analysis. Figures are prepared with pyEMMA 2.5.7 (www.emma-project.org).

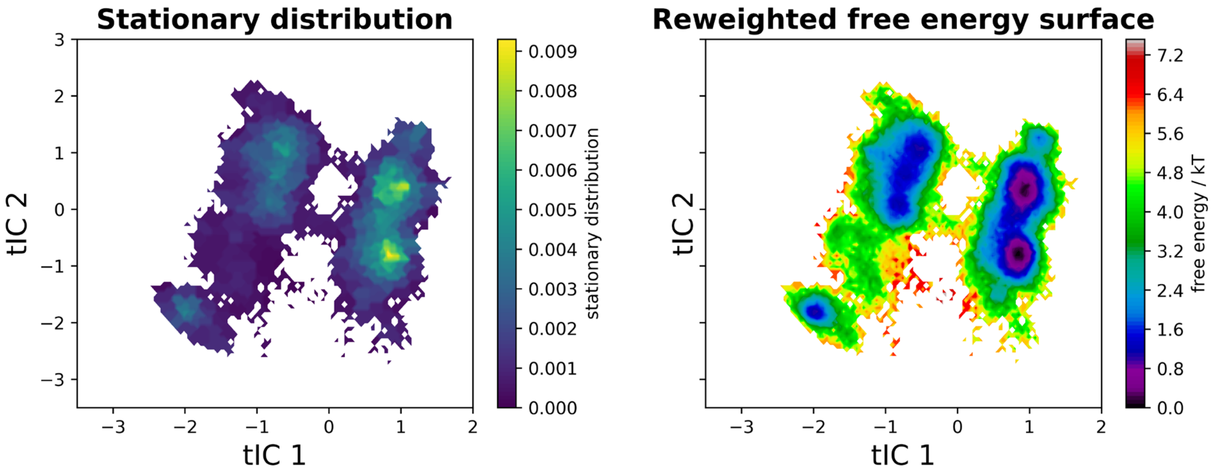

Figure S7. The stationary distribution of the sST2 loop conformations and the reweighted free energy surface of sST2 using a lag time of 80 steps. Figures are prepared with pyEMMA 2.5.7 (www.emma-project.org).

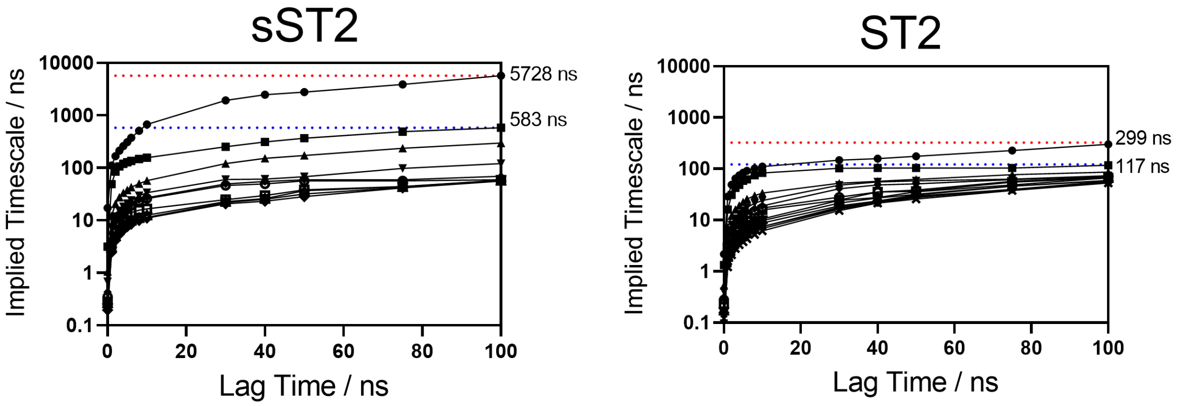

Figure S8. Implied timescales of the loop dynamics estimated from the MSM analysis using the ensemble of sST2 and ST2. The end points of the slowest implied timescales are shown in the blue dot lines and annotated. Figures are prepared using GraphPad Prism 8.

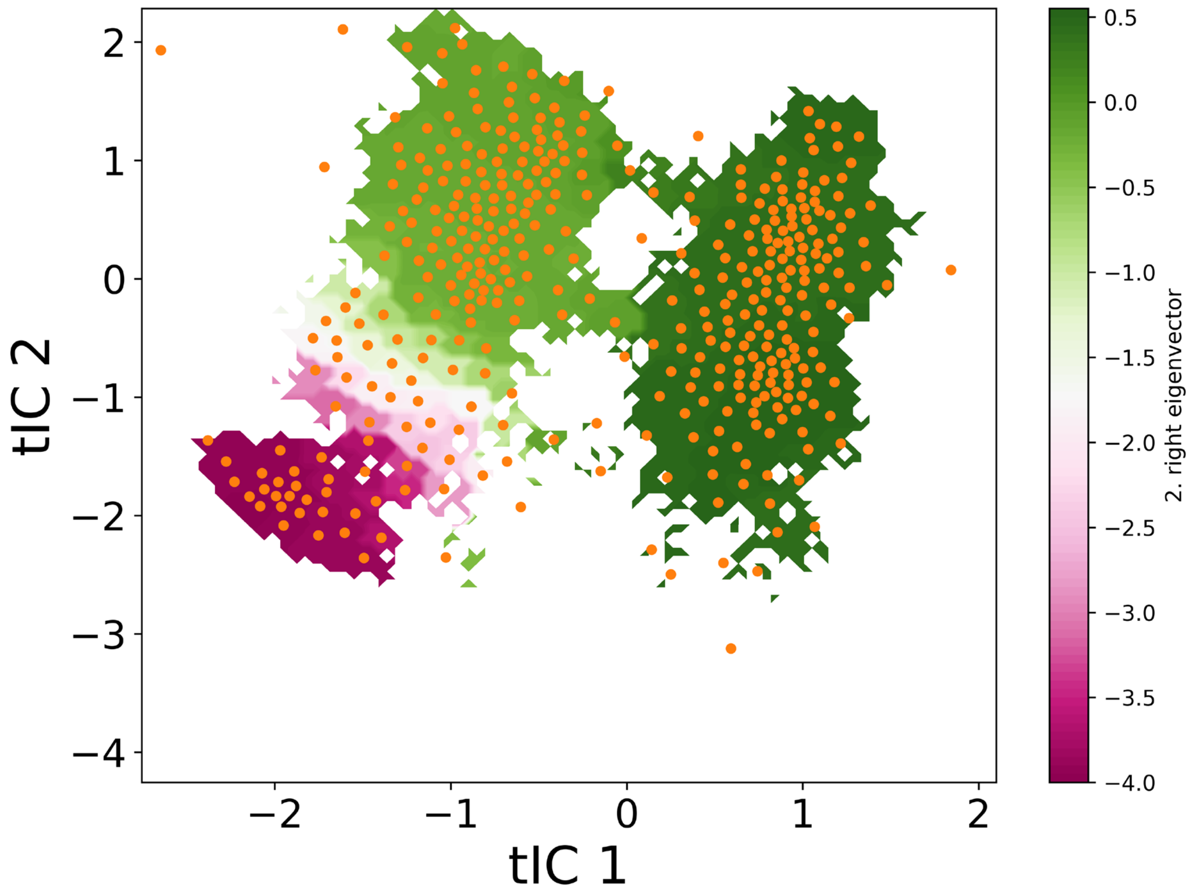

Figure S9. The microstates (orange dots) and the transition flux of the second eigenvector (the slowest implied timescale) in the MSM of the sST2 loop motions. The positive and negative values of the eigenvector correspond to in- and out-flux in reference to the stationary distribution. Figures are prepared with pyEMMA 2.5.7 (www.emma-project.org).

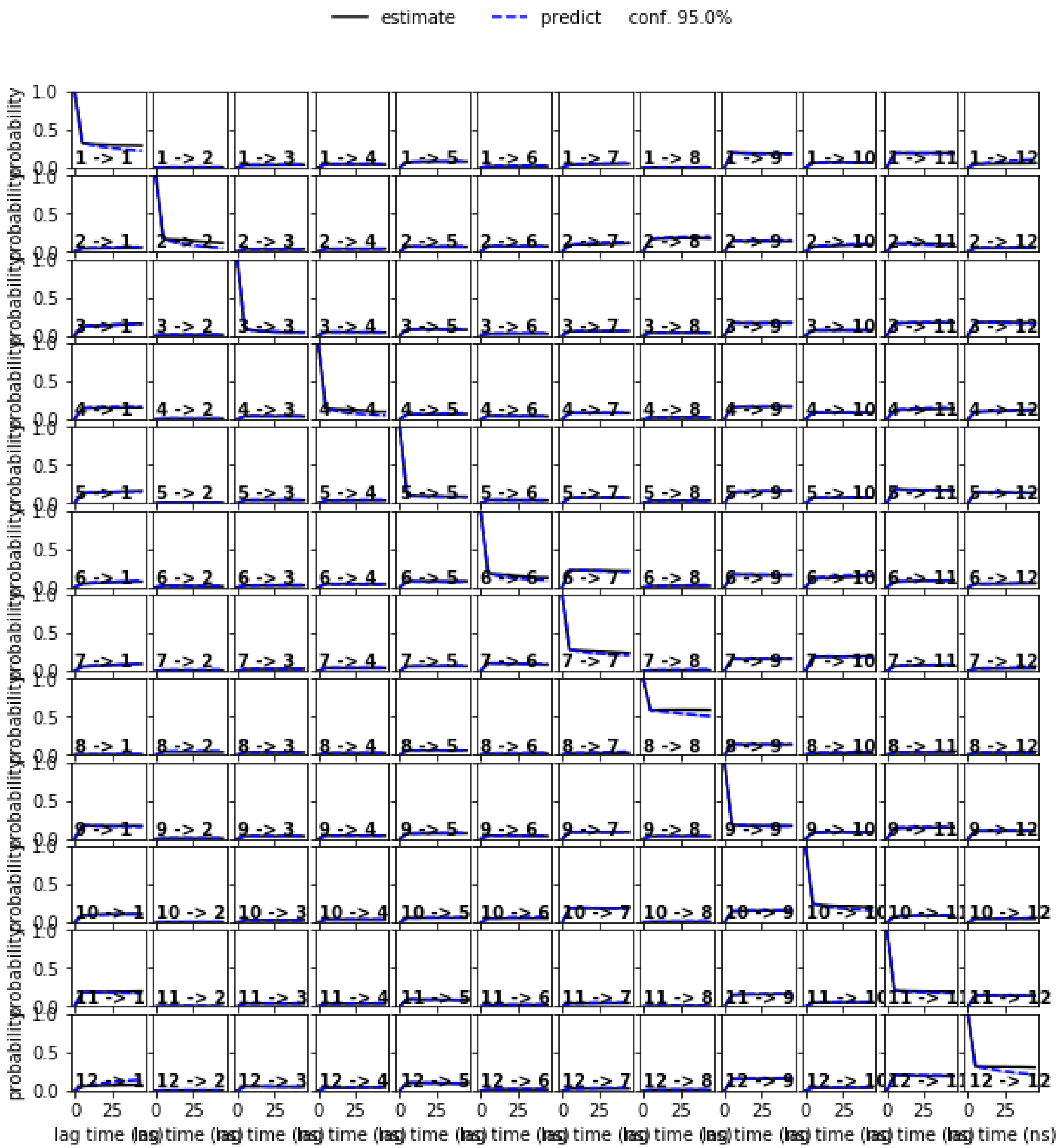

Figure S10. The CK test of the sST2 ensemble based on 12 macrostates. Figures are prepared with pyEMMA 2.5.7 (www.emma-project.org).

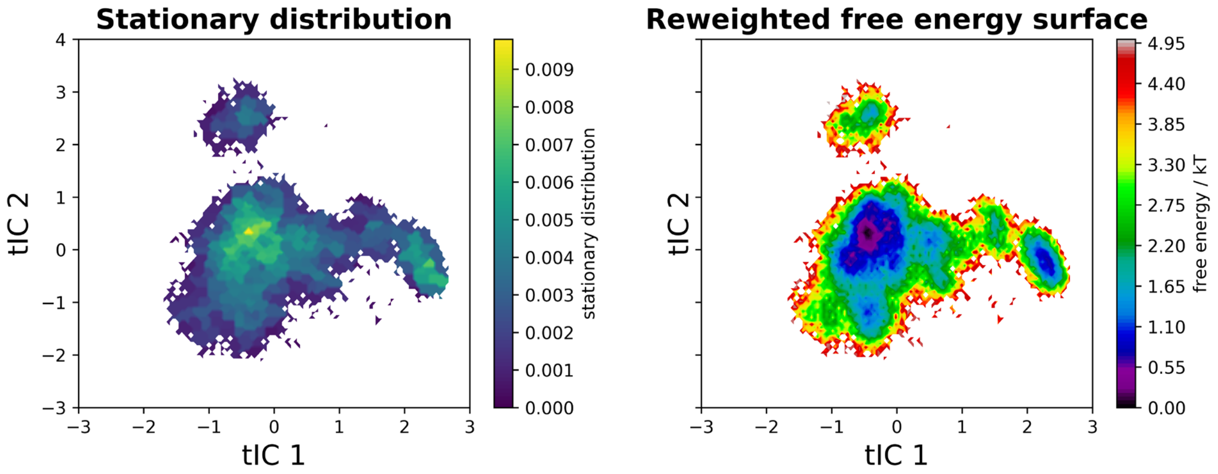

Figure S11. The stationary distribution of the ST2 loop conformations and the reweighted free energy surface of ST2 using a lag time of 80 steps. Figures are prepared with pyEMMA 2.5.7 (www.emma-project.org).

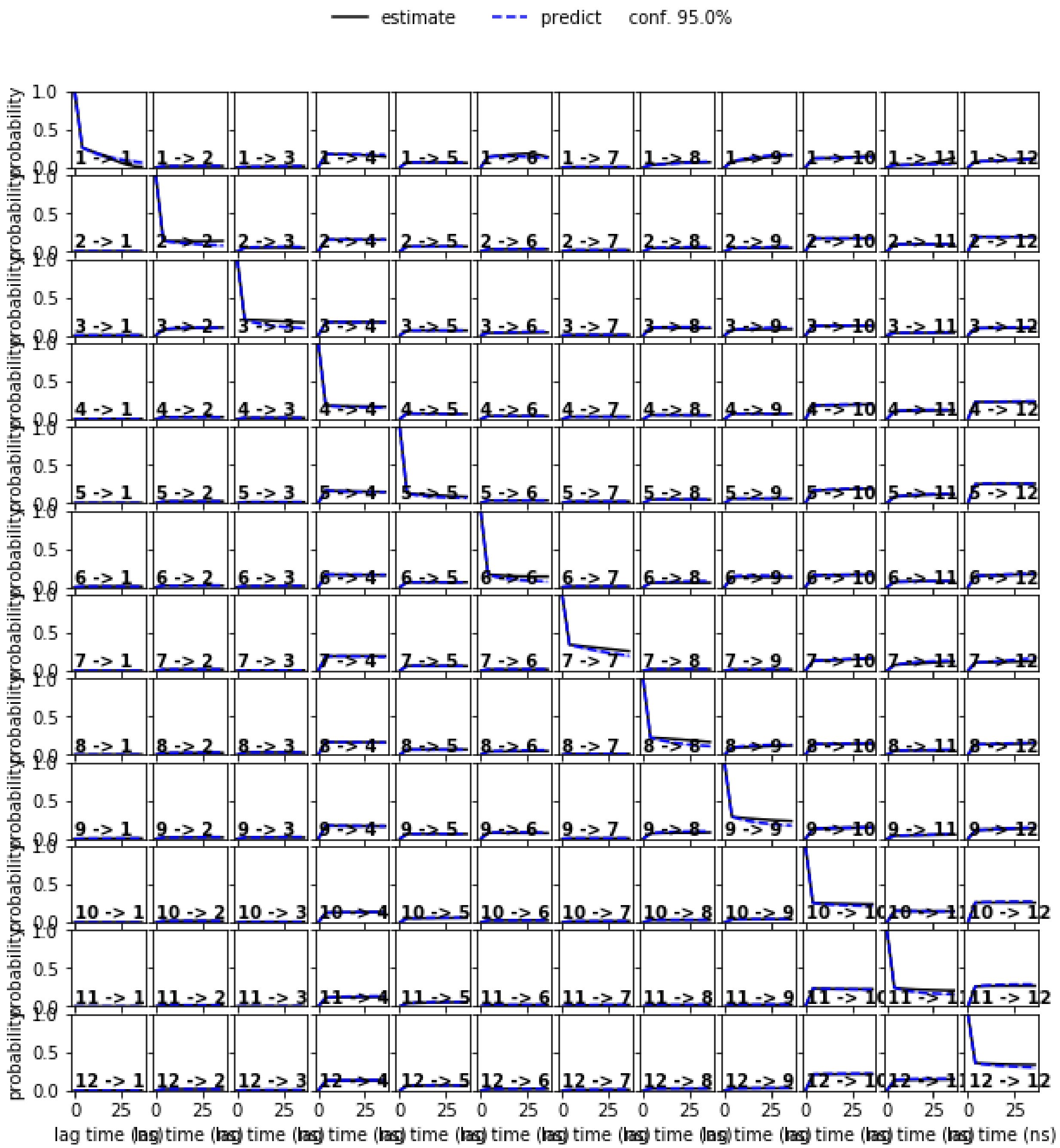

Figure S12. The CK test of the ST2+sST2 ensemble based on 12 macrostates. Figures are prepared with pyEMMA 2.5.7 (www.emma-project.org).

PC	Proportion of Variance (%)
1	47.17
2	30.64
3	10.95
4	6.27
5	2.19
6	1.57
7	0.53
8	0.36
9	0.19
10	0.06
11	0.04
12	0.02

Table S1. Proportion of variance of each principal component in PCA.
